# Associations of dietary diversity with depressive symptoms, anxiety symptoms and their co-occurrence among reproductive-age women in three low- and middle-income countries: a cross-sectional analysis

**DOI:** 10.3389/fpubh.2026.1955678

**Published:** 2026-10-20

**Authors:** Jing Fang, Liying Huang, Ningyuan Fang

**Affiliations:** 1 Department of General Practice, Ren Ji Hospital, Shanghai Jiao Tong University School of Medicine, Shanghai, China; 2 Punan Hospital of Pudong New Area, Shanghai, China

**Keywords:** anxiety symptoms, co-occurring depressive and anxiety symptoms, depressive symptoms, dietary diversity, minimum dietary diversity for women (MDD-W)

## Abstract

**Background:**

Depressive and anxiety symptoms are common among women of reproductive age in low- and middle-income countries (LMICs). Whether dietary diversity is associated with depressive symptoms, anxiety symptoms, and their co-occurrence in this population remains insufficiently characterized.

**Objectives:**

To examine the associations between minimum dietary diversity for women (MDD-W) and depressive symptoms, anxiety symptoms, and co-occurring depressive and anxiety symptoms among reproductive-age women in three LMICs.

**Methods:**

A cross-sectional analysis was conducted using Demographic and Health Surveys (DHS) data from Zambia, Mozambique, and Nepal, including 34,576 women aged 15–49 years. Depressive and anxiety symptoms were assessed using the Patient Health Questionnaire-9 (PHQ-9) and Generalized Anxiety Disorder-7 (GAD-7), respectively. Minimum dietary diversity for women (MDD-W) was classified as inadequate (<5 food groups) versus adequate (≥5 food groups). Survey-design-based multivariable logistic regression models accounting for DHS sampling weights, clustering, and stratification were used to estimate odds ratios (ORs) and 95% confidence intervals (CIs). Subgroup analyses assessed potential effect modification. Sensitivity analyses were performed by modeling dietary diversity as a continuous score and by repeating the primary analyses separately by country and after excluding currently pregnant women.

**Results:**

The weighted prevalence of depressive symptoms, anxiety symptoms, and co-occurring depressive and anxiety symptoms was 5.7%, 6.4%, and 3.9%, respectively. In fully adjusted models, meeting MDD-W was associated with lower odds of depressive symptoms (OR = 0.69, 95% CI: 0.58–0.82), anxiety symptoms (OR = 0.63, 95% CI: 0.54–0.73), and co-occurring symptoms (OR = 0.58, 95% CI: 0.47–0.72). Food-group analyses showed inverse associations with depressive symptoms, anxiety symptoms, and co-occurring symptoms for other vegetables, pulses, eggs, flesh foods, other fruits, and dairy products, whereas dark green leafy vegetables were positively associated with all three mental health measures. Subgroup analyses indicated possible heterogeneity by residence, wealth status, and marital status for selected measures. Sensitivity analyses showed generally inverse associations across countries, although the magnitude and statistical significance varied; spline analyses indicated nonlinear associations, and results were similar after excluding currently pregnant women.

**Conclusion:**

Among reproductive-age women in three LMICs, meeting MDD-W was associated with lower odds of depressive symptoms, anxiety symptoms, and their co-occurrence. Longitudinal and intervention studies are needed to clarify the temporal and causal relationships between dietary diversity and mental health symptoms.

## Introduction

Depressive symptoms, anxiety symptoms, and their co-occurrence are highly prevalent in low- and middle-income countries (LMICs) and contribute significantly to the global burden of disease and disability (1, 2). LMICs account for 80.19% of the global burden of major depressive disorder and women exhibit a higher prevalence of mental health problems than men (3). Women of reproductive age are particularly vulnerable because they fulfill diverse roles within both familial and societal contexts and frequently encounter a range of risk factors, including economic pressures, caregiving responsibilities and social inequalities (4). These challenges increase their vulnerability to mental health issues and hinder their access to timely interventions (5). Therefore, identifying modifiable factors associated with women’s mental health is important.

In recent years, nutrition has been recognized as an important modifiable factor influencing mental health (6). Adequate and balanced dietary intake helps maintain neurotransmitter synthesis, regulate inflammatory responses and preserve gut-brain axis function (7). Moreover, nutrients such as protein, B vitamins, vitamin D, magnesium, zinc, selenium, iron, calcium, and omega-3 fatty acids have been associated with depressive and anxiety symptoms (8). However, existing studies have focused on single nutrients or overall dietary patterns and have been conducted primarily in high-income country populations, whereas evidence specifically targeting women in LMICs remains relatively limited (9). In many low-income households, diets are monotonous and predominantly cereal-based, with poor nutritional quality and insufficient intake of vegetables, fruit, and animal-source foods, thereby raising the risk of micronutrient deficiencies (10). MDD-W is a food-group diversity indicator developed specifically for women of reproductive age. It is defined as the consumption of at least five of ten food groups during the previous day and night and indicates a greater likelihood of micronutrient adequacy, rather than providing a comprehensive assessment of overall diet quality (11, 12). MDD-W was selected because it is appropriate for women of reproductive age and was assessed consistently across the DHS datasets.

Previous single-country studies using MDD-W or related dietary diversity scores have reported inverse associations with depressive and anxiety symptoms among reproductive-age women in Nepal and other female populations (13–15). A recent meta-analysis also found an inverse association between dietary diversity and depressive symptoms, although the included evidence was cross-sectional and showed substantial heterogeneity (16). However, it remains unclear whether dietary diversity is associated simultaneously with depressive symptoms, anxiety symptoms, and their co-occurrence across distinct LMIC settings. Country-specific variation and associations with individual food groups also remain insufficiently characterized.

Therefore, this study examined the associations of minimum dietary diversity for women (MDD-W) with depressive symptoms, anxiety symptoms, and co-occurring depressive and anxiety symptoms among women of reproductive age based on the Demographic and Health Surveys (DHS) data from Zambia, Mozambique, and Nepal.

## Methods

### Study population

A cross-sectional analysis was conducted using data from Zambia DHS 2024, Mozambique DHS 2022–2023, and Nepal DHS 2022. According to the World Bank country and lending group classification, Mozambique is classified as a low-income economy, whereas Nepal and Zambia are classified as lower-middle-income economies. These countries were therefore considered LMIC settings in this study (17). The DHS program was designed to collect information on maternal and child health and socio-demographic characteristics at the household level in more than 90 LMICs (18). DHS adopts a 2-stage sampling approach: (1) selecting survey clusters stratified by geographic region and urban or rural residence; and (2) within each cluster 20 to 30 households are randomly sampled for interviews. A total of 34,576 women with complete mental health and dietary information were included from three LMICs across South Asia and Sub-Saharan Africa in our study. All included women also had complete data for the covariates used in the regression models. The detailed flowchart of participant selection is shown in Supplementary Figure S1. The publicly available population data used in this study have been reviewed and approved by the Institutional Review Board of ICF International and written consent was obtained from the participants (19).

### Dietary diversity measure

The dietary information was assessed using dietary diversity score for women based on dietary intake in the past day and night (12). This was the standardized dietary assessment available across the three DHS datasets. In a 24 h food consumption recall 10 food groups were assessed: (1) grains (white/pale starchy roots, tubers, and plantains); (2) pulses (beans, peas, and lentils); (3) nuts and seeds; (4) dairy (milk, cheese, yogurt, other milk products); (5) flesh foods (meat, fish, poultry and liver/organ meats); (6) eggs; (7) dark green leafy vegetables; (8) vitamin A rich fruits and vegetables; (9) other vegetables and (10) other fruits (14, 20). The dietary diversity score ranged from 0 to 10. Details are shown in Supplementary Table S1. Higher scores indicate greater diversity, as more food groups were reported as being consumed. Based on the dietary diversity score, MDD-W was defined as a dichotomous indicator: women with a score below five food groups were classified as having inadequate dietary diversity, while those consuming five or more food groups were considered to have adequate dietary diversity.

### Mental health measures

Women aged between 15 and 49 years were assessed for depressive symptoms by the Patient Health Questionnaire (PHQ-9). It is a nine-item self-report tool that evaluates the presence of depressive symptoms during the previous 2 weeks, with a total score that ranges from 0 to 27. Each item is scored from 0 to 3. In our study, depressive symptoms were defined as a PHQ-9 score ≥ 10 (21, 22). The country-specific weighted Cronbach’s alpha coefficients were 0.81 in Zambia, 0.92 in Mozambique, and 0.86 in Nepal.

The Generalized Anxiety Disorder Scale (GAD-7) was used to measure anxiety symptoms. Another self-reported scale consists of seven items that evaluate the occurrence and severity of generalized anxiety symptoms during the previous 2 weeks. Total scores range from 0 to 21, and a score ≥10 was used to define anxiety symptoms consistently across the three countries, based on the commonly used cutoff (23). The country-specific weighted Cronbach’s alpha coefficients were 0.79 in Zambia, 0.92 in Mozambique, and 0.86 in Nepal. Because the DHS datasets did not include clinical diagnostic interviews, the criterion validity of the PHQ-9 and GAD-7 could not be independently assessed in this study. Women who met both the PHQ-9 and GAD-7 thresholds were classified as having co-occurring depressive and anxiety symptoms.

### Covariates

Based on prior evidence, the following covariates were included: demographic characteristics (age, marital status, residence), socioeconomic status (education level, wealth status, employment status, media exposure), reproductive history (number of living children, history of child death, current pregnancy status), and environmental factors (source of drinking water, type of sanitation facility) (14, 20, 24, 25).

### Statistical analysis

Continuous variables were described as weighted means with standard deviations (SDs), and categorical variables were described as frequencies with weighted percentages. Survey-design-based logistic regression models were used to examine the associations between dietary diversity and each mental health measure. Model 1 was adjusted for country fixed effects only; Model 2 adjusted for demographic and socioeconomic factors; and Model 3 additionally adjusted for reproductive and environmental factors. Country fixed effects were included in all models. All models incorporated the DHS individual sampling weight (v005/1,000,000), primary sampling units defined by combining country with v021, and sampling strata defined by combining country with v022. No additional rescaling of the sampling weights was performed after pooling the three datasets.

To further explore the specific components of dietary diversity that may be associated with mental health measures, separate analyses were conducted for each of the 10 individual food groups. We treated each food group as a binary variable (indicating consumption versus non-consumption) and examined its association with depressive symptoms, anxiety symptoms, and co-occurring symptoms in a separate fully adjusted survey-weighted logistic regression model. The food groups were not mutually adjusted for one another. To account for multiple comparisons, *p* values from the food-group analyses were adjusted using the Benjamini–Hochberg false discovery rate procedure.

Finally, stratified analyses were conducted to observe potential moderating effects across different subgroups, including (1) age groups (15–24, 25–34, and 35–49 years), (2) marital status (married versus single), (3) place of residence (urban versus rural), (4) educational levels (no education, primary, secondary, and higher), (5) wealth index (poor, middle, and rich) and (6) history of child death (yes versus no). Effect modification was tested using multiplicative interaction terms (P for interaction).

Sensitivity analyses were performed in three ways. First, the primary analyses were repeated separately in each country. Second, the dietary diversity score was modeled as a continuous variable using natural cubic splines, and the spline models were compared with linear models using Wald tests to assess nonlinearity. Finally, the primary models were repeated after excluding currently pregnant women. All data analyses were completed using R statistical software version 4.5.2. Odds ratios (ORs) and 95% confidence intervals (CIs) were reported. All statistical tests were two-sided, and *p*-values < 0.05 were considered statistically significant.

## Results

### Descriptive data

Table 1 summarizes the baseline characteristics of 34,576 reproductive-aged women (15–49 years) across Zambia (40.4%), Mozambique (38.2%), and Nepal (21.5%). The overall prevalence of depressive symptoms, anxiety symptoms, and co-occurring symptoms was 5.7%, 6.4%, and 3.9%, respectively. Socio-demographically, participants were fairly evenly distributed across the three age groups. The majority were married (62.0%), resided in rural areas (50.0%), reported media exposure (60.9%), and had at least primary education (81.6%). Additionally, 45.3% of women belonged to the rich wealth quintile, and 43.3% were employed. Regarding reproductive and environmental factors, 37.8% of women had more than two living children, 12.1% had a history of child death and 6.3% were pregnant. Most households had access to improved drinking water (70.6%) and sanitation facilities (53.9%). Supplementary Figure S2 illustrates that women with adequate dietary diversity consumed a broader range of food groups more frequently, particularly fruits, vegetables, legumes, eggs, dairy, and flesh foods.

**Table 1:** Baseline characteristics of women.

Variables	Study population (*N* = 34,576)
Depressive symptoms, *n* (%)	
No	32,966 (94.3)
Yes	1,610 (5.7)
Anxiety symptoms, *n* (%)	
No	32,858 (93.6)
Yes	1718 (6.4)
Co-occurring symptoms, *n* (%)	
No	33,583 (96.1)
Yes	993 (3.9)
Age group (years), *n* (%)	
15–24	13,125 (37.6)
25–34	11,184 (33.2)
35–49	10,267 (29.2)
Marital status, *n* (%)	
Single	13,305 (38.0)
Married	21,271 (62.0)
Residence status, *n* (%)	
Rural	18,734 (50.0)
Urban	15,842 (50.0)
Education level, *n* (%)	
Higher	1,633 (5.0)
Secondary	13,274 (38.0)
Primary	13,645 (38.6)
None	6,024 (18.4)
Media exposure, *n* (%)	
No	12,963 (39.1)
Yes	21,613 (60.9)
Wealth status, *n* (%)	
Poor	12,766 (35.7)
Middle	6,724 (19.0)
Rich	15,086 (45.3)
Employment, *n* (%)	
No	18,590 (56.7)
Yes	15,986 (43.3)
Total number of children, *n* (%)	
≤2	21,368 (62.2)
>2	13,208 (37.8)
Children died, *n* (%)	
No	30,142 (87.9)
Yes	4,434 (12.1)
Pregnant, *n* (%)	
No	32,395 (93.7)
Yes	2,181 (6.3)
Drinking water source, *n* (%)	
Improved	25,109 (70.6)
Unimproved	7,920 (24.2)
Other	1,547 (5.2)
Sanitation facility, *n* (%)	
Improved	18,134 (53.9)
Unimproved	13,596 (38.9)
Other	2,846 (7.2)
Country, *n* (%)	
Mozambique	13,183 (38.2)
Nepal	7,442 (21.5)
Zambia	13,951 (40.4)

All *n* values are unweighted raw counts of the study sample. All percentages are weighted using the standardized DHS individual sampling weight to represent the target population of reproductive aged women.

### Results of regression analyses

Table 2 presents the survey-weighted logistic regression analyses examining the associations between MDD-W and the odds of depressive symptoms, anxiety symptoms, and co-occurring symptoms. Overall, meeting MDD-W was associated with lower odds of depressive symptoms, anxiety symptoms, and co-occurring symptoms across all models. In the fully adjusted model (Model 3), the ORs were 0.69 (95% CI: 0.58–0.82) for depressive symptoms, 0.63 (95% CI: 0.54–0.73) for anxiety symptoms, and 0.58 (95% CI: 0.47–0.72) for co-occurring symptoms. The associations remained inverse and statistically significant across Models 1–3.

**Table 2:** Associations between MDD-W and depressive symptoms, anxiety symptoms, and co-occurring symptoms.

Model	Depressive symptoms	Anxiety symptoms	co-occurring symptoms
	OR (95% CI)	OR (95% CI)	OR (95% CI)
Model 1	0.62 (0.53, 0.73)	0.59 (0.51, 0.69)	0.52 (0.43, 0.64)
Model 2	0.70 (0.59, 0.83)	0.64 (0.55, 0.74)	0.59 (0.48, 0.73)
Model 3	0.69 (0.58, 0.82)	0.63 (0.54, 0.73)	0.58 (0.47, 0.72)

Model 1 was adjusted for country fixed effects only.

Model 2 was adjusted for age, marital status, place of residence, education level, media exposure, household wealth status, and employment status.

Model 3 was further adjusted for number of living children, experience of child mortality, pregnant status, source of drinking water, and sanitation facility.

All models accounted for DHS sampling weights, country-specific primary sampling units, and country-specific sampling strata. OR, odds ratio; CI, confidence interval.

Table 3 shows the associations between individual food groups and mental health measures in fully adjusted models. After FDR correction, consumption of other vegetables, pulses, eggs, flesh foods, other fruits, and dairy products was inversely associated with depressive symptoms, anxiety symptoms, and co-occurring symptoms. Vitamin A-rich fruits and vegetables were inversely associated with anxiety symptoms and co-occurring symptoms, but not depressive symptoms. Other vegetable consumption was inversely associated with depressive symptoms (OR = 0.55, 95% CI: 0.48–0.64), anxiety symptoms (OR = 0.65, 95% CI: 0.56–0.75), and co-occurring symptoms (OR = 0.47, 95% CI: 0.39–0.57). In contrast, dark green leafy vegetable consumption was positively associated with all three measures (depressive symptoms: OR = 1.42, 95% CI: 1.20–1.69; anxiety symptoms: OR = 1.28, 95% CI: 1.11–1.47; co-occurring symptoms: OR = 1.67, 95% CI: 1.37–2.02). Grains/roots/tubers were inversely associated only with depressive symptoms, while nuts and seeds were inversely associated with anxiety symptoms and co-occurring symptoms but not depressive symptoms.

**Table 3:** Associations between individual food groups and depressive symptoms, anxiety symptoms, and co-occurring symptoms.

Food groups	Depressive symptoms	FDR-adjusted *p*	Anxiety symptoms	FDR-adjusted *p*	Co-occurring symptoms	FDR-adjusted *p*
	OR (95% CI)		OR (95% CI)		OR (95% CI)	
Other vegetables	0.55 (0.48, 0.64)	<0.01	0.65 (0.56, 0.75)	<0.01	0.47 (0.39, 0.57)	<0.01
Pulses	0.62 (0.53, 0.72)	<0.01	0.62 (0.53, 0.72)	<0.01	0.49 (0.41, 0.59)	<0.01
Eggs	0.67 (0.54, 0.82)	<0.01	0.66 (0.54, 0.81)	<0.01	0.58 (0.44, 0.75)	<0.01
Flesh foods	0.75 (0.65, 0.87)	<0.01	0.74 (0.64, 0.85)	<0.01	0.63 (0.52, 0.76)	<0.01
Other fruits	0.66 (0.54, 0.79)	<0.01	0.67 (0.57, 0.79)	<0.01	0.60 (0.47, 0.76)	<0.01
Dairy products	0.77 (0.63, 0.94)	0.01	0.71 (0.61, 0.84)	<0.01	0.68 (0.54, 0.85)	<0.01
Vitamin-A rich fruits and vegetables	0.82 (0.67, 1.01)	0.06	0.70 (0.58, 0.83)	<0.01	0.73 (0.57, 0.93)	0.01
Grains/roots/tubers	0.59 (0.39, 0.90)	0.02	0.75 (0.49, 1.14)	0.19	0.91 (0.56, 1.48)	0.70
Nuts and seeds	0.85 (0.69, 1.06)	0.17	0.75 (0.60, 0.92)	<0.01	0.64 (0.49, 0.84)	<0.01
Dark green leafy vegetables	1.42 (1.20, 1.69)	<0.01	1.28 (1.11, 1.47)	<0.01	1.67 (1.37, 2.02)	<0.01

All models are fully adjusted for age group, marital status, urban/rural residence, education level, media exposure, household wealth status, employment status, number of living children, experience of child death, pregnancy status, drinking water source, sanitation facility, country fixed effects. All analyses accounted for DHS individual sampling weights, country-specific primary sampling units, and country-specific sampling strata. OR, odds ratio; CI, confidence interval. *p* values were adjusted across all 30 food group–mental health measure comparisons using the Benjamini–Hochberg false discovery rate procedure.

Table 4 presents the subgroup analyses exploring the potential modifying effects of the associations between MDD-W and the three mental health measures. In subgroup analyses, effect estimates were generally inverse across the examined subgroups. Significant interactions were observed for residence with depressive symptoms (P for interaction = 0.02) and co-occurring symptoms (P for interaction = 0.04), wealth status with depressive symptoms (P for interaction = 0.03), and marital status with co-occurring symptoms (P for interaction = 0.04). Sensitivity analyses showed generally inverse country-stratified estimates, although their magnitude and statistical significance varied across countries (Supplementary Figure S3). The spline analyses indicated nonlinear associations between the dietary diversity score and depressive symptoms, anxiety symptoms, and co-occurring symptoms (all P for nonlinearity <0.001; Supplementary Figure S4). The results remained similar after excluding currently pregnant women (Supplementary Table S2).

**Table 4:** Subgroup analyses of the association between minimum dietary diversity for women and depressive symptoms, anxiety symptoms, and co-occurring symptoms.

Variable	Count	Depressive symptoms	Anxiety symptoms	Co-occurring symptoms	P for interaction		
		OR (95% CI)	OR (95% CI)	OR (95% CI)	Depressive	Anxiety	Co-occurring
Age group							
15–24	13,125	0.87 (0.65, 1.15)	0.79 (0.58, 1.06)	0.80 (0.55, 1.17)	0.14	0.16	0.19
25–34	11,184	0.59 (0.46, 0.77)	0.57 (0.44, 0.75)	0.46 (0.32, 0.66)			
35–49	10,267	0.65 (0.50, 0.85)	0.60 (0.47, 0.75)	0.55 (0.39, 0.76)			
Marital status							
Single	13,305	0.76 (0.59, 0.99)	0.72 (0.54, 0.96)	0.79 (0.56, 1.09)	0.28	0.19	0.04
Married	21,271	0.65 (0.53, 0.80)	0.59 (0.49, 0.71)	0.49 (0.37, 0.64)			
Residence status							
Rural	18,734	0.82 (0.64, 1.05)	0.69 (0.55, 0.88)	0.69 (0.50, 0.97)	0.02	0.09	0.04
Urban	15,842	0.63 (0.50, 0.78)	0.61 (0.50, 0.74)	0.53 (0.41, 0.68)			
Education level							
No education	6,024	0.74 (0.53, 1.03)	0.53 (0.39, 0.73)	0.49 (0.31, 0.77)	0.53	0.76	0.65
Primary	13,645	0.71 (0.54, 0.94)	0.70 (0.54, 0.91)	0.58 (0.40, 0.84)			
Secondary	13,274	0.66 (0.50, 0.86)	0.66 (0.51, 0.85)	0.69 (0.51, 0.93)			
Higher	1,633	0.73 (0.38, 1.39)	0.79 (0.42, 1.49)	0.56 (0.24, 1.29)			
Wealth index							
Poor	12,766	0.80 (0.60, 1.06)	0.69 (0.52, 0.91)	0.67 (0.44, 1.02)	0.03	0.30	0.18
Middle	6,724	0.79 (0.56, 1.11)	0.67 (0.47, 0.96)	0.59 (0.38, 0.92)			
Rich	15,086	0.62 (0.49, 0.78)	0.61 (0.49, 0.76)	0.55 (0.42, 0.71)			
Child death							
No	30,142	0.68 (0.57, 0.82)	0.63 (0.53, 0.74)	0.58 (0.46, 0.72)	0.10	0.14	0.05
Yes	4,434	0.77 (0.50, 1.20)	0.68 (0.45, 1.02)	0.66 (0.39, 1.12)			

Counts are unweighted. Adequate minimum dietary diversity for women (MDD-W; consumption of at least five of ten food groups) was compared with inadequate dietary diversity. Adjusted odds ratios (ORs) and 95% confidence intervals (CIs) were estimated using survey-weighted logistic regression accounting for DHS sampling weights, clustering, and stratification. Models included country fixed effects and the fully adjusted covariate set, excluding the corresponding stratification variable. P for interaction was obtained from Wald tests of the MDD-W-by-subgroup interaction term.

## Discussion

Using large-sample DHS data from three low- and middle-income countries, our study found that meeting MDD-W was associated with lower odds of depressive symptoms, anxiety symptoms, and co-occurring symptoms among women of reproductive age. At the food-group level, other vegetables, eggs, flesh foods, other fruits, and dairy products were inversely associated with depressive symptoms, anxiety symptoms, and co-occurring symptoms, whereas dark green leafy vegetables were positively associated with all three measures. Subgroup analyses indicated possible heterogeneity by residence, wealth status, and marital status for selected measures. Country-stratified estimates were generally directionally consistent but varied in magnitude, and the continuous-score analyses suggested nonlinear associations.

Our findings are consistent with previous research conducted in both LMICs and developing countries (14, 26–28). For example, a cross-sectional study of 48,637 women in China showed that a higher dietary diversity score was associated with lower odds of depressive symptoms (13), whereas our study provides a more comprehensive assessment by also assessing anxiety symptoms and co-occurring depressive and anxiety symptoms. This inverse association is further supported by other retrospective cohort studies (15, 29). Individuals in the adequate dietary diversity group had a higher consumption proportion of fruits, vegetables, eggs and other such foods.

At the food group level, our findings were partly consistent with previous reviews suggesting that higher intake of vitamin A-rich fruits and vegetables and dietary fiber is associated with lower levels of depressive and anxiety symptoms (30, 31). In contrast to our findings, a previous study in single country reported no significant association between dark green leafy vegetable consumption and depressive symptoms (OR = 0.91, 95% CI: 0.72–1.15) or anxiety symptoms (OR = 0.84, 95% CI: 0.68–1.04) (14). The three countries differ in their food systems, dietary practices, food availability, and socioeconomic conditions, which may contribute to the variation in estimates across countries (Supplementary Figure S3). Differences in dietary assessment methods, including the use of a single 24-h dietary recall, may also partly explain the discrepant findings (11, 31). Residual confounding, reverse causation, and chance findings due to multiple comparisons cannot be excluded.

Subgroup analyses indicated possible heterogeneity in the associations between MDD-W and selected mental health measures according to place of residence, wealth status, and marital status. The stronger inverse associations observed among urban and wealthier women for depressive symptoms, and among married women for co-occurring depressive and anxiety symptoms, may reflect differences in household resources, food access, and broader social context. Recent studies have reported links between dietary diversity, food security, and mental health in women and LMIC populations (14, 16, 32). Differences in survey timing, food availability, and dietary assessment may also contribute to variation across settings (11). However, these factors were not directly assessed in the present study, and these explanations remain speculative. Given the multiple subgroup comparisons and the cross-sectional design, these findings should be interpreted cautiously and considered exploratory.

Several possible explanations might partly explain the observed association between dietary diversity and mental health. First, dietary diversity is a fundamental component of a healthy dietary pattern. At the nutritional level, a more diverse diet is generally a proxy for improved micronutrient adequacy and a broader intake of protein, B-group vitamins, minerals, antioxidants, and essential fatty acids, all of which are relevant to neurotransmitter synthesis, synaptic signaling, and affective regulation (11, 33). In addition, dietary diversity may be associated with differences in gut microbial composition and gut–brain signaling, which are increasingly implicated in mental health (34). In LMIC settings, dietary diversity may further reflect better food security and overall living conditions, both of which are closely related to women’s psychological well-being (10). However, food insecurity was not directly assessed in our study and may represent an unmeasured source of residual confounding. Although causal mechanisms cannot be established in this cross-sectional study, these pathways provide a biologically and socially plausible interpretation of our findings.

However, several limitations should also be acknowledged. First, the cross-sectional design limits causal inference, making it impossible to determine the temporal direction of the observed associations. Future prospective cohort studies and randomized controlled trials are needed to explore causal relationships. Second, all data on dietary diversity, depressive symptoms, and anxiety symptoms relied on self-report measures, introducing the potential for recall and reporting bias; moreover, the PHQ-9 and GAD-7 thresholds do not represent clinical diagnoses. Dietary diversity was assessed using a single 24-h recall, which may not reflect habitual intake, day-to-day or seasonal variation, or portion size. Moreover, MDD-W is an indicator of food-group diversity intended to reflect the likelihood of micronutrient adequacy, rather than a comprehensive measure of overall diet quality. Although current pregnancy status was adjusted for and the sensitivity analysis excluding pregnant women yielded similar results, MDD-W was not specifically developed to assess micronutrient adequacy during pregnancy. Therefore, the interpretation of MDD-W in pregnant women should be made cautiously. Third, although our study controlled for multiple potential confounders, the possibility of residual confounding from other relevant variables could not be excluded. For instance, experiences of traumatic events such as natural disasters, menopausal status, and attitudes toward help-seeking were not measured, limiting a comprehensive understanding of all potential contributors to mental health measures. Household food insecurity was not measured and may therefore represent an unmeasured source of residual confounding. Finally, the large number of food-group and subgroup comparisons means that some findings may have occurred by chance and should therefore be interpreted cautiously.

## Conclusion

In this cross-sectional study of women aged 15–49 years in three LMICs, meeting MDD-W was associated with lower odds of depressive symptoms, anxiety symptoms, and their co-occurrence. Longitudinal and intervention studies are needed to clarify the temporal and causal relationships between dietary diversity and women’s mental health before dietary diversity can be recommended as a mental health promotion strategy.

## Data Availability

Publicly available datasets were analyzed in this study. This data can be found at The DHS Program Data Repository: https://dhsprogram.com/data/available-datasets.cfm, Datasets analyzed from Zambia DHS 2024, Mozambique DHS 2022–23, and Nepal DHS 2022. Access is available upon registration and approval of a data access request through The DHS Program. No accession numbers apply.
