# Supplementary material for: Associations of dietary diversity with depressive symptoms, anxiety symptoms and their co-occurrence among reproductive-age women in three low- and middle-income countries: a cross-sectional analysis
Source: Front Public Health. 2026 Oct 20;14:1955678. doi: 10.3389/fpubh.2026.1955678 (PMC13727591; doi:10.3389/fpubh.2026.1955678)
Supplement: Supplementary file 1 [file Supplementary_file_1.docx]

**Figure S1**. Study Population.

**Figure S2.** Food consumption proportions by dietary diversity status

**Figure S3**. Country-stratified associations between MDD-W and depressive symptoms, anxiety symptoms, and co-occurring symptoms.

**Figure S4.** Associations between dietary diversity score and depressive symptoms, anxiety symptoms, and co-occurring symptoms.
