# Supplementary material for: Associations of dietary diversity with depressive symptoms, anxiety symptoms and their co-occurrence among reproductive-age women in three low- and middle-income countries: a cross-sectional analysis
Source: Front Public Health. 2026 Oct 20;14:1955678. doi: 10.3389/fpubh.2026.1955678 (PMC13727591; doi:10.3389/fpubh.2026.1955678)
Supplement: Supplementary file 2 [file Supplementary_file_2.docx]

**Table S1. Food groups for calculating minimum dietary diversity (MDD).**

| Food groups | Items |
| --- | --- |
| Grains/roots/tubers | Grains (Rice, Wheat, Maize, Kawin), Roots and Tubers (bread, rice) |
| Pulses | Beans, peas, lentils |
| Dairy products | Milk, Yoghurt, Cheese |
| Flesh foods | Meat, fish, poultry and liver/organ meats |
| Dark green leafy vegetables | Spinach, green chili, ladies’ finger, pumpkin leaves, sweet potato leaves |
| Vitamin-A rich fruits and vegetables | Ripen mango, ripen papaya, carrot, pumpkin, sweet potatoes, green mango, green papaya and vegetables |
| Others vegetables | Any type of vegetables |
| Other fruits | Any type of fruits |
| Nuts and seeds | Any type of nuts and seeds |
| Eggs | - |

**Note:** Consumption of foods from at least 5 of the following 10 food groups during the previous day or night was classified as meeting MDD-W.

**Table S2.** **Sensitivity analysis excluding currently pregnant women**

|  | Depressive symptoms | Anxiety symptoms | Co-occurring symptoms |
| --- | --- | --- | --- |
|  | OR (95% CI) | OR (95% CI) | OR (95% CI) |
| Model 1 | 0.62 (0.53, 0.73) | 0.61 (0.52, 0.70) | 0.52 (0.43, 0.65) |
| Model 2 | 0.69 (0.58, 0.83) | 0.65 (0.55, 0.76) | 0.59 (0.47, 0.73) |
| Model 3 | 0.68 (0.57, 0.82) | 0.64 (0.55, 0.76) | 0.58 (0.47, 0.73) |

**Model 1** was adjusted for country fixed effects only.

**Model 2** was adjusted for age group, marital status, place of residence, education level, media exposure, household wealth status, and employment status.

**Model 3** was further adjusted for number of living children, experience of child death, source of drinking water, and sanitation facility.

All models accounted for sampling weights, clustering, and stratification. OR, odds ratio; CI, confidence interval. All P values were <0.001.
